# Evaluating the mobility potential of antibiotic resistance genes in environmental resistomes without metagenomics

**DOI:** 10.1038/srep35790

**Published:** 2016-10-21

**Authors:** Katariina Pärnänen, Antti Karkman, Manu Tamminen, Christina Lyra, Jenni Hultman, Lars Paulin, Marko Virta

**Affiliations:** 1 University of Helsinki, Department of Food and Environmental Sciences, POB 56, 00014 University of Helsinki, Finland; 2 University of Helsinki, Department of Biosciences, POB 65, 00014 University of Helsinki, Finland; 3 ETH Zürich, Department of Environmental Systems Science, Universitätstrasse 16, 8092 Zürich, Switzerland; 4 Eawag, Swiss Federal Institute of Aquatic Science and Technology, Dübendorf, Überlandstrasse 611, 8600 Dübendorf, Switzerland; 5 University of Helsinki, Department of Food Hygiene and Environmental Health, POB 66, 00014 University of Helsinki, Finland; 6 University of Helsinki, Institute of Biotechnology, DNA Sequencing and Genomics Laboratory, POB 56, 00014 University of Helsinki, Finland

## Abstract

Antibiotic resistance genes are ubiquitous in the environment. However, only a fraction of them are mobile and able to spread to pathogenic bacteria. Until now, studying the mobility of antibiotic resistance genes in environmental resistomes has been challenging due to inadequate sensitivity and difficulties in contig assembly of metagenome based methods. We developed a new cost and labor efficient method based on Inverse PCR and long read sequencing for studying mobility potential of environmental resistance genes. We applied Inverse PCR on sediment samples and identified 79 different MGE clusters associated with the studied resistance genes, including novel mobile genetic elements, co-selected resistance genes and a new putative antibiotic resistance gene. The results show that the method can be used in antibiotic resistance early warning systems. In comparison to metagenomics, Inverse PCR was markedly more sensitive and provided more data on resistance gene mobility and co-selected resistances.

In the grimmest predictions, we are entering a pre-antibiotic era, where even minor infections can be fatal because antibiotics cannot be effectively used due to increased antibiotic resistance^1^. However, antibiotics and antibiotic resistance genes (ARGs) are ancient, having many ecological functions in natural environments, and date back to pre-clinical era^2^,^3^. Therefore, resistance genes are found everywhere, including environments that have not been exposed to anthropogenic impact^4^,^5^,^6^.

It is assumed that antibiotic resistance, including resistance to clinically relevant antibiotics, has originated in the environment^7^. The main driving force behind spreading resistance between the environmental and clinical bacteria is horizontal gene transfer mediated by mobile genetic elements (MGEs), such as transposons, plasmids and lysogenic bacteriophages^8^,^9^,^10^. Because the majority of ARGs of environmental bacteria are not associated with MGEs, they are not easily transferred to clinically relevant bacteria^3^,^5^. Therefore, the majority of antibiotic resistance genes of environmental bacteria do not cause a threat, since dissemination of the non-mobile genes is unlikely. However, the environmental resistome is under selective pressure from human activities^7^, which increases the proportion of ARGs associated with MGEs making the environmental resistome more mobile^11^. Consequently, the mobility potential of environmental ARGs has been raised as in important aspect of resistome studies, since mobile ARGs can potentially be transferred to clinically relevant bacteria, and therefore the genes cause an increased risk to human health^12^.

Several strategies are used for studying ARGs and associated MGEs in both clinical and non-clinical environments including cultivation, PCR–based methods and metagenomics. The most comprehensive approach for studying antibiotic resistance in the environment is metagenomics, where total DNA derived from all the organisms in a sample is sequenced^13^,^14^,^15^,^16^. Currently the most commonly used sequencing technologies produce short reads, which are assembled into longer contigs^17^. If the assembled contigs are long enough to resolve the genetic environment of the ARGs, the mobility potential can be assessed based on identifying MGE associated genes. The benefit of metagenomics is that no prior knowledge of the ARG sequences is needed, whereas PCR based methods require this information for primer design. However, there are two major challenges in applying metagenomics for studying environmental ARGs. First, in most natural environments the relative abundance of ARGs is low, which increases the requirement for sequencing depth and costs substantially^18^. Second, the assembly of short reads into longer contigs can be challenging for samples from diverse environments^17^. New sequencing technologies such as PacBio RS^19^ and Oxford Nanopore MinION^20^ produce long reads making assembly easier. Despite advancements, even with the newest long read technologies large sequencing effort is needed to detect rare genes. Due to these limitations, there is a need for new methods not relying on metagenomics for studying antibiotic resistance’s mobility potential in the environment.

To overcome limitations of metagenomics in studying MGE association of ARGs, we have developed a method which combines Inverse PCR^21^, (IPCR) with long read sequencing to study the genetic context of ARGs. The method was applied to sediment samples collected below an open-cage fish farm^22^,^23^. In IPCR, up- and downstream regions of the ARG are enriched using primers specific to the ARG and the resulting amplicons are sequenced with long read sequencing technology (Figs 1 and 2). Following sequencing, genes flanking the ARGs are identified, which permits determining the potential MGE association of each investigated ARG. We verified that IPCR is more suitable for studying ARG mobility by comparing the resulting IPCR amplicons to long contigs obtained with metagenomic sequencing from the same samples. IPCR proved to be markedly more sensitive as well as a more cost and labor efficient method for studying antibiotic resistance in environmental samples.

## Results

A total of 7034 CCS (circular consensus sequence) reads were obtained from the PacBio SMRT cell sequencing of IPCR amplicons, out of which 6108 matched *sul1* and 626 *tetM* genes. 5546 reads passed quality filtering (mean PHRED score 41, length distribution 113-4511 and mean length 2423). The reads were clustered with USEARCH and the mean cluster size was 52 ± 24 using 97.5% CI. The CCSs formed 79 clusters with consensus sequences longer than 1500 bp.

Several different *sul1* containing mobile genetic elements were identified within the IPCR consensus sequences (Fig. 3): a bacteriophage, a Tn*21* transposon with class I integrons, a type In4 class I integron and two other uncharacterized types of class I integrons. Consensus sequences with In0/In2/In5 class I integron^24^ had an *aadA*1 aminoglycoside resistance gene cassette, *tniB∆* and *tniA* genes, IS*1326* insertion sequence containing *istA* and *istB∆*, as well as Tn*21* transposon associated genes *tnpR* and *tnpM* (Fig. 3). These consensus sequences were 99–100% identical to integrons of clinical isolates (i.e. *Pseudomonas aeruginosa* CP011317.1). The consensus sequences with In4 type class I integron had an IS*1600* insertion sequence *tnpA* transposase as well as *sul1*, *orf5* and *orf6* (Fig. 3). The In4 sequence was 100% identical to several In4 class I integrons of clinically relevant isolates (i.e. *Pseudomonas aeruginosa* AP017302.1). One of the class I integrons had an aminoglycoside resistance gene *aadA*5 (99% identity, score 1657, *Pseudomonas aeruginosa* GU250441.1).

An MGE containing *sul1* and *orf5* had bacteriophage associated genes encoding protein D and V, M15 peptidase and a hypothetical protein. The genes were closely related (96% sequence identity) to genes from sulfur reducing bacteria (i.e. *Geobacter sulfurreducens* CP010430.1 and *Geoalkalibacter subterraneus* CP010311.1) infected with bacteriophages and did not have hits to any clinical bacterial isolates in the nr database.

In addition to the In0/In2/In5 and In4 integrons, an unknown type class I integron with a putative novel bla_oxa_ type β-lactamase gene and *ereA* erythromycin resistance gene were identified using IPCR (Fig. 3). The newly discovered ARG had low similarity to known bla_oxa_ genes from non-pathogenic environmental bacteria (i.e. class D β-lactamase gene, *Synechococcus* sp. NKBG15041c, WP_024546971.1, score 363, amino acid identity 77%)

Only two different types of mobile genetic elements, a Tn*916* type transposon and an MGE with the IS*CR20* transposase gene, were identified in the consensus sequences with *tetM* (Fig. 4). The Tn*916* type transposon consensus sequences had high similarity (99–100% sequence identity) to Tn*916* transposons sequenced from clinical isolates (i.e. *Streptococcus suis*, FM252032.1 and *Streptococcus pneumoniae* CP001033.1). Also the IS*CR20 tnpA* gene was 99% identical to genes in NCBI nr nucleotide database (i.e. *Escherichia coli* strain R170 plasmid pRZA92, NG_040885.1, score 859). Both Tn*916* orf6 and *orf9* and the IS*CR20 tnpA* gene were also identified in clone sequences.

Illumina MiSeq metagenome sequencing of the sediment samples collected under fish farm cages produced over 25 million reads and 13 Gbps of data after quality filtering (Table 1). The quality filtered individual reads were mapped against the Comprehensive Antibiotic Resistance gene Database, CARD^25^. The relative abundances of the unassembled metagenomic reads containing *sul1* and *tetM* compared to 16S rRNA gene were between 10^−3^ and 10^−4^ (Table 1). Only 8 of the reads mapped to *sul1* and 44 to *tetM* (Table 1).

Assembly of reads was done using three different assemblers, metaSPADES^26^, MEGAHIT^27^, and IDBA-UD^28^, to minimize bias caused by assembler. Assembly statistics are given in Table 2. None of the assemblies contained contigs with *sul1*. However, with *tetM* two different MGEs (Tn*916* and Tn*5397*) were identified from the assemblies. MEGAHIT and metaSPADES assemblies both produced three contigs containing *tetM*. Neither assemblies had *tetM* contigs longer than 3 kbps. IDBA-UD produced four contigs containing *tetM*, with one 4596 bp long contig and three contigs between 1.8 and 2.8 kbps. In addition to the *tetM* contigs, four contigs containing other ARGs associated with MGEs not containing *sul1* and *tetM* genes were identified from the metagenomic assemblies. The MGE associated ARGs encoded resistance to tetracyclines or aminoglycosides. Altogether, the number of all ARG associated MGE contigs was lower in the metagenomic libraries comparing to the *sul1* and *tetM* IPCR libraries despite the fact that the IPCR libraries had just two target ARGs. IPCR was more sensitive than metagenomics. The sensitivity limit of non-nested IPCR was determined to be approximately 10^−8^ copies of target gene per 16S rRNA gene, whereas for metagenomes the abundance limit for obtaining assembled contigs with target genes was approximately 10^−3^ target genes per 16S rRNA gene.

## Discussion

MGE association of ARGs increases the likelihood of gene transfer and it might facilitate the dissemination of environmental resistance genes to clinically relevant pathogenic bacteria^12^. We developed a new cost and labor efficient method based on IPCR^21^ and long read sequencing to evaluate the potential of horizontal transfer of environmental ARGs. IPCR was applied to study the dissemination potential associated with *sul1* and *tetM* resistance genes found in the sediment underneath an open cage fish farm located in the Baltic Sea. The dissemination potential was assessed by identifying possible MGE association of the *sul1* and *tetM* genes. IPCR’s sensitivity was verified by metagenome sequencing and assembly of the metagenomic reads from the same samples.

By combining IPCR with long read sequencing we effectively identified multiple different MGEs associated with both *sul1* and *tetM* genes. In total the IPCR sequencing reads formed 79 clusters using 90% sequence identity cut off. With IPCR, just for *sul1*, several different MGEs were identified despite the low abundance of *sul1* in studied samples^22^. In contrast, conclusions could not be made about the mobility potential of *sul1* based on the metagenomes, since no contigs containing *sul1* were formed despite the use of three different assemblers. In the metagenomic libraries only two different MGEs could be identified from the assembled contigs for either of the genes in contrast to the roughly ten different MGEs discovered using ARG targeted IPCR. Assessing the transfer potential of ARGs using metagenomics would require a several fold increase in the sequencing depth, which would also increase the sequencing costs and the required computing time.

Metagenomics does not require prior knowledge of ARGs and is in a sense an open-ended method for studying the resistome. However, the difficulties caused by sequencing depth requirements and contig assembly in environments with low ARG abundances have been described previously^15^,^29^. By using metagenomics no contigs containing ARGs could be assembled from a lake metagenome with an ARG abundance comparative to our study (10^−3^ ARGs/16S rRNA gene)^15^. Also with deep sequencing of sewage treatment plant metagenomes, the assembly success of ARG associated MGEs was limited likely due to the mosaic structure of MGEs which contain conserved genes in different genetic contexts making contigs truncated at regions which are hard to resolve for the assembly algorithms^29^. Because of the technical complications related to metagenomics studies, new methods suitable for early warning systems for emerging mobile antibiotic resistance genes are of great importance. Using an enrichment step such as IPCR coupled with long read sequencing is an effective approach to target sequencing effort to known ARGs and the unknown ARG flanking genes.

IPCR evades challenges posed by sequencing depth and contig assembly that are typically associated with metagenomics^17^,^18^. This was achieved by enriching target DNA with IPCR and subsequently sequencing the amplicons with PacBio^19^, which produces long sequencing reads. However, using IPCR for enrichment can cause a bias because of sequence specific primers. We observed that the transposon Tn*5397* was not detected in the IPCR sequences even though it was discovered in the metagenomic libraries, which might be due to using primers that do not amplify the *tetM* gene variant of Tn*5397* or inadequate sequencing depth of the *tetM* PacBio IPCR ampicons. There is no reason why several sets of primers or nested PCR could not be used with IPCR, since multiple PCRs can be done with one sample preparation, and no primer specific barcodes are needed for sequencing since the PCR amplicon sequences differ due to different primers. In contrast, obtaining several kilobasepair contigs needed to assess the MGE association of *sul1* and *tetM* genes would require a massive increase metagenomic sequencing depth and costs, since the relative abundance of the *sul1* and *tetM* target genes is low (10^–3^–10^−4^ relative to the 16S rRNA gene) and the genes are in many different types of MGEs. Even with state of the art metagenomic methods, there are challenges in assembling and identifying ARGs and MGEs in environments with low ARG abundances.

Unlike the metagenomic libraries, IPCR could be used to study the MGEs associated with both *sul1* and *tetM*. All of the *sul1* and *tetM* IPCR consensus sequences obtained from the fish farm sediment samples had high similarity to known mobile genetic elements or related genes, such as transposases, suggesting that there is an increased potential of horizontal transfer. The *sul1* gene was associated with class I integrons of In0/In2/In5 and In4 types as well as two other class I integrons. Moreover, the In0/In2/In5 and In4 were identical to class I integrons isolated from clinical pathogens such as *P. aeruginosa*. *tetM* was associated with a Tn*916* transposon identical to ones found in clinical isolates of pathogenic tetracycline resistant strains.

IPCR was also used to detect co-selected ARGs in the integrons and transposons. *ereA* and *aadA* genes encoding resistances to erythromycin and aminoglycosides were found in the same MGEs as *sul1*, but were not found in the metagenome contigs. Intriguingly, aminoglycosides and erythromycin have never been used in the sampled fish farm, but are commonly used to treat infections in humans. This underlines that our method is capable of finding associations not only to MGEs but also to other ARGs, which are co-selected. In this case, co-selection of *ereA* and *aadA* is worrisome, since they encode resistance to clinically relevant antibiotics.

Co-selection of a new putative bla_oxa_ type β-lactam resistance gene was discovered in a class I integron concensus sequence together with the *sul1*, *orf5*, *qacE∆1* and *ereA* genes. The bla_oxa_ had low similarity to a class D β-lactamase of marine cyanobacteria *Synechococcus* sp. NKBG15041c, WP_024546971.1. Also other database matches of the putative novel bla_oxa_ were to environmental bacteria (such as *Acinetobacter baumannii*). The bla_oxa_ gene had been captured by a class I integron MGE causing a possible risk due to the increased likelihood of horizontal transfer^12^. The discovery of a novel MGE associated bla_oxa_ using IPCR shows that finding novel ARGs captured by MGEs is possible without costly and computationally intensive metagenomic studies.

In addition to the known class I integrons and transposons identified from the MGEs associated with su11, novel MGE contexts of *sul1* and *tetM* were discovered, which shows that the ARGs are acquired on new MGEs. Lysogenic bacteriophage genes were associated with the *sul1* and *orf5*. The bacteriophage genes were highly similar to those found in non-pathogenic sulphur reducing environmental bacteria such as *Geobacter sulfurreducens*. Bacteriophages have recently been suggested to play a role in the dissemination of ARGs especially between different and even distant environments^8^. In addition to bacteriophages, an IS*CR20* transposase gene^30^ was found with *tetM* using IPCR. IS*CR20* has not been previously described to be associated with *tetM*. Finding new MGE associations for *su11* and *tetM* using IPCR, shows that IPCR can be applied for studying previously unknown MGEs and ARG connections.

Our method based on IPCR and PacBio SMRT cell sequencing for studying horizontal gene transfer potential of antibiotic resistance genes is useful for environmental resistome studies and proved to be markedly more sensitive as well as more cost and labor efficient that metagenomics. Less sequencing effort and computing time was needed since primers are used to target the sequencing effort to the ARGs’ genetic environment. Additionally, the bioinformatics workflow is simple as no CPU demanding assembly is needed. In addition to expected known MGEs, such as clinical transposases and integrons, we also dicovered MGEs and a novel mobile ARG. Since long read sequencing is becoming more available to researchers with Minion sequencing^20^, IPCR could be used to quickly assess the mobility of environmental ARGs, including low abundance genes and resistances of emerging concern.

## Materials and Methods

### Sediment sampling

Sediment samples were collected in 2006, 2009 and 2013 from a fish farm located in the northern Baltic Sea during sampling campaigns of previous studies^22^,^23^. The sediments were stored in −20 °C before DNA extraction. The farm raises European white fish (*Coregonus lavaretus*) and rainbow trout (*Oncorhynchus mykiss*) and has a history of antibiotic use. Top layer of the sediments was sampled using a Limnos sediment probe (Limnos Ltd., Finland). Specific details of the samples and sampling campaigns have been described previously^22^,^23^.

### DNA isolation, restriction enzyme digestion and circularization

DNA was extracted from 0.5 g of sediment using PowerSoil^®^ DNA isolation kit (MO BIO Laboratories, USA) according to the manufacturer’s instructions. DNA was extracted from three biological replicate samples of sediment collected from three different locations within the fish farm in 2013 and of one replicate per year from the 2006 and 2009 samples. The DNA isolations were pooled together. A second set of DNA extractions was done from the same samples and the isolated DNA was used for metagenomic sequencing. The sediments were stored in −20 °C before DNA extraction.

The isolated sediment DNA from different years was pooled and concentrated using AmiconUltra 30 K centrifugation units (Millipore, Merck, USA). DNA was digested with HincII, NcoI, XbaI, XhoI, EcoRI and PstI restriction enzymes according to manufacturer’s instructions (Thermo Scietific, USA). A total of 15 μg of DNA was used for all the digestions.

5 μg of DNA digested with HincII or EcoRI was circularized with 25 U of T4 ligase (Thermo Scientific, USA) in a final volume of 1 ml of 1X T4 ligation buffer. The reaction was carried out in 22 °C for 1 hour. The ligation mixture was concentrated and purified using Amicon Ultra 30 K centrifugation units (Millipore, Merck, USA).

Also a pooled circularization reaction was done using DNA digested with HincII, NcoI, XbaI, Xho and PstI. 5 μg of the DNA was circularized using T4 ligase with 45 U of T4 ligase (Thermo Scientific, USA), 10% v/v of PEG 4000 (Thermo Scientific, USA) and in a final volume of 5 ml of 1X T4 ligation buffer. The ligation was carried out in 17 °C over night. The ligated DNA was concentrated and purified using AMPure XP SPRI paramagnetic beads (Agencourt, Beckman Coulter Inc., USA) according to manufacturer’s instructions and eluted in sterile MilliQ H2O. DNA concentrations for unpooled and pooled circularization reactions were measured using Qubit^®^ 3.0 fluorometer (Thermo Scientific, USA).

### IPCR

**Figure 1:**
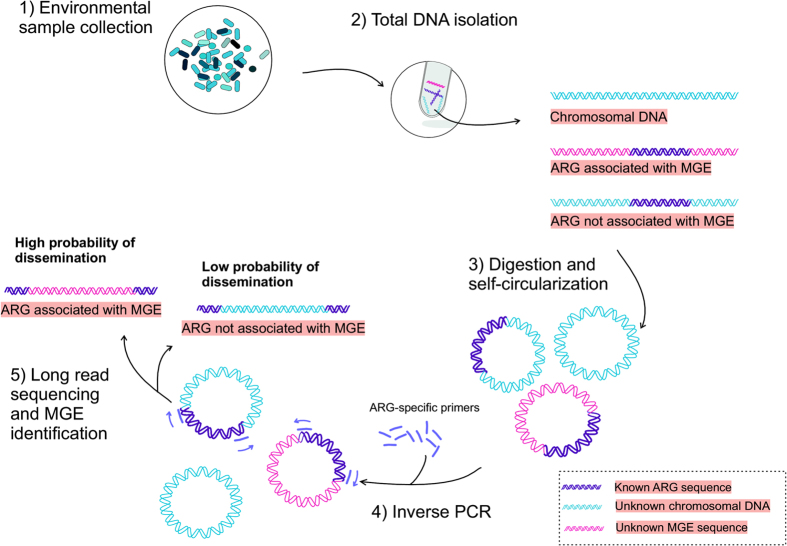
Protocol for using IPCR to evaluate the horizontal gene transfer potential of ARGs in the environment. After sample collection (1) and total DNA extraction (2), the DNA is digested with restriction enzymes and resulting fragments are self-ligated into circular DNA molecules (3). DNA flanking the ARG is amplified with IPCR using ARG targeting primers (4). The amplicons are sequenced using long read sequencing with PacBio SMRT cell technology and the ARG associated MGEs are identified (5).

**Figure 3:**
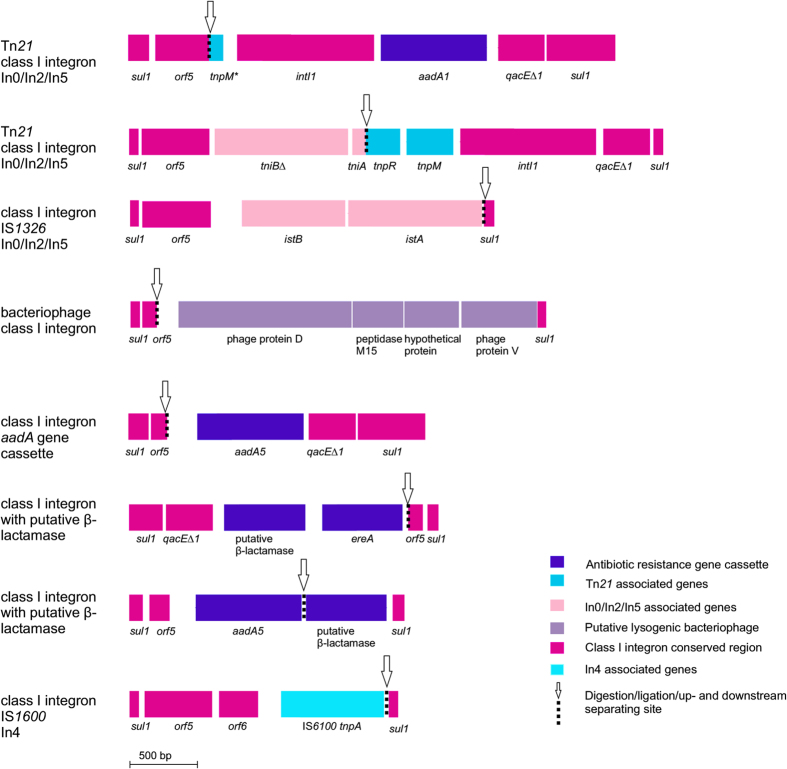
Mobile genetic elements containing *sul1* resistance genes identified from IPCR consensus sequences. The restriction and ligation site, where the MGE sequence is discontinuous, is marked with an arrow. The genes and open reading frames are oriented as in Fig. 2 in relation to the MGE sequence.

**Figure 4:**
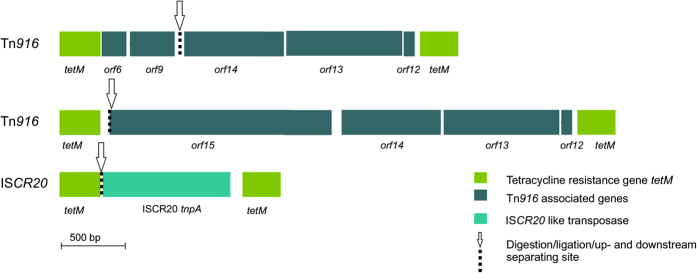
t*etM* resistance gene containing mobile genetic elements identified from IPCR consensus sequences. The restriction and ligation site, where the MGE sequence is discontinuous, is marked with an arrow. The genes and open reading frames are oriented as in Fig. 2 in relation to the MGE sequence.

IPCR for *tetM* was done using self-circularized DNA digested with either HincII or EcoRI (Fig. 1). IPCR was done with 5–15 ng of DNA, 0.4 U of Phusion DNA polymerase (Thermo Scientific, USA), 0.5 μM concentration of each *tetM* inv FW and *tetM* inv RV primer (Table 3), 200 μM concentration of each dNTP in a final volume of 20 μl of 1X GC buffer. The cycling conditions were as follows: 98 °C for 30 s and 40 cycles of 98 °C for 10 s, 59 °C for 30 s, 72 °C for 2 min, and a final extension at 72 °C for 10 min.

IPCRs for *tetM* and *sul1* were done with pooled digestions using the self-circularized DNA digested with HincII, NcoI, XbaI, Xho and PstI. DNA was amplified in reactions with 10–20 ng of DNA, 1.25 U PrimeSTAR GXL DNA-polymerase (TaKaRa BIO Inc., Japan), 200 μM concentration of each dNTP, 0.2 μM concentration of *sul1* inv FW and *sul1* inv RV (reverse complemented, Pei *et al.* 2006) or *tetM* inv FW and *tetM* inv RV primers (Table 3) and a final volume of 50 μl of 1X reaction buffer. The cycling conditions were as follows: 30 cycles of 98 °C for 10 s, 60 °C for 15 s and 68 °C for 4 min. The PCR products were purified using Ampure XP (Agencourt, Beckman Coulter Inc., USA). DNA concentration was measured using Qubit fluorometer (Thermo Scientific, USA).

### Non-nested IPCR sensitivity determination

The minimum copy number of target DNA fragments for IPCR and PacBio sequencing was determined by performing a serial dilution of 10 kb and 15 kb fragments of *Escherichia coli* genomic DNA in Salmon Sperm DNA (Invitrogen, USA). Dilutions of 1 to 10^−6^ were done corresponding to 10^8^–10^2^ copies of target genomic DNA. The DNA was ligated using T4 ligase (Thermo Scientific, USA) and purified using Ampure XP (Agencourt, Beckman Coulter Inc., USA) as described previously. IPCR amplification of the genomic *E. coli* fragment was done with *E. coli* specific primers and one round of IPCR as described previously using PrimeSTAR GXL DNA-polymerase (TaKaRa BIO Inc., Japan). The PCR product was run on 1% agarose gel stained with ethidium bromide. Visible bands on the gel were interpreted as being successful and had adequate DNA quantities for PacBio sequencing. For 10 kb fragments the sensitivity limit was 100 copies per reaction and 1000 copies for the 15 kb fragments. The target gene abundance compared to 16S rRNA gene was calculated assuming one copy of 16S rRNA gene per genome and an average genome size of 4.7 Mb.

### *tetM* IPCR clone library construction

10 ng of the *tetM* IPCR -product was ligated into pUC19 plasmid cut with SmaI using T4 ligase (Thermo Scientific, USA) according to manufacturer’s instructions. The construct was transformed into Subcloning Efficiency™ DH5α™ competent *E.coli* cells (Thermo Scientific, USA) according to manufacturer’s instructions. Constructs were purified using QIAprep Spin Miniprep plasmid isolation kit (Qiagen, Germany). The *tetM* IPCR inserts were amplified using pUC19 vector primers and purified as previously described using Ampure XP SPRI paramagnetic beads (Agencourt, Beckman Coulter Inc., USA).

### Nested IPCR

Since DNA yield of the first round of IPCR was low, nested IPCR was performed to obtain more DNA for sequencing. Nested IPCR primers amplifying a shorted amplicon than the *sul1* inv FW and RV primers were designed for *sul1* using PrimerBLAST26 primer design program^31^. The primers obtained with PrimerBLAST were reverse complemented to reverse the PCR amplification direction. Nested IPCR was done as described above with 10 ng of template DNA amplified with *sul1* inv FW and RV primers using the *sul1* nest primers (Table 3). The PCR products were purified using Ampure XP SPRI paramagnetic beads (Agencourt, Beckman Coulter Inc., USA) as described previously. DNA concentration was measured using Qubit fluorometer (Thermo Scientific, USA).

### PacBio sequencing, quality filtering and clustering of the IPCR amplicons

All of the IPCR products were pooled for sequencing 1.2 μg of *sul1* nested IPCR products, 150 ng of *sul1* and 350 ng of *tetM* IPCR products and the *tetM* cloned inserts. The IPCR products were sequenced with PacBio RS II (Pacific Biosciences of California, Inc., USA). Sequencing was done at the Institute of Biotechnology (University of Helsinki, Finland) using one SMRT Cell^®^ (Pacific Biosciences of California, Inc., USA) without adding barcodes. The sequences have been deposited to SRA under project PRJNA326347.

The sequences were divided into three groups based on BLAST searches against representative *sul1* and *tetM* sequences retrieved from Genbank nucleotide database. The used representative gene sequence for *sul1* was from *Vibrio chlorerae* (KP076293.1, 4305–5144 bp). For *tetM* three sequences were used; *Chlamydia trachomatis* (CVNZ01003190.1, 25–999 bp), *Streptococcus pneumoniae* (CVOF01000004.1, 55153–57072 bp) and *Enterococcus faecalis* (AJ585077.1, 1–1417 bp). BLAST search was done using the megaBLAST algorithm^32^ using default parameters.

Quality of the PacBio reads of insert was analyzed using FastQC (available from http://www.bioinformatics.babraham.ac.uk/projects/fastqc/). Reads of insert were quality filtered with USEARCH^33^ -fastq_filter command with -fastq_maxee_rate 0.01 and -fastq_minlen 100 parameters.

Clustering was done using UCLUST^33^ -cluster_fast command and with several different identity percentages. 90% identity was chosen for obtaining consensus sequences. Consensus sequences shorter than 1500 bp were removed from further analysis.

### Metagenomic DNA sequencing, sequence quality filtering, read assembly and contig annotation

Paired-end sequencing (2 × 300 bp) was done using Illumina Miseq platform at the Institute of Biotechnology (University of Helsinki, Finland) from the same sediment samples that were used for IPCR. The sequences have been submitted to the MG-RAST database under project accession number 9119.

Adapters were removed using Cutadapt^34^ and the reads were quality trimmed using StreamingTrim^35^ with the option -q 20. Quality trimmed reads were mapped using BWA^36^ with the bwa mem -command with default parameters against the CARD antibiotic resistance database^25^. Bacterial 16S rRNA gene reads were retrieved and calculated using metaxa2^37^.

Assemblies of the reads were done using three different assemblers. MEGAHIT^27^ assembly was done using the –presets meta-sensitive option. IDBA-UD^28^ run was done with the -mink 50 option. metaSPADES16 v3.7.1^26^ was run with the parameters –p meta -k 55,77,99. Contigs shorter than 1500 bp were discarded from further analysis. BLAST searches of the resulting contigs were done against representative *sul1* and *tetM* sequences as described previously.

### Chimera checking, gene annotation and identification of mobile genetic elements

Manual annotation of the IPCR consensus sequences and metagenome contigs containg *sul1* or *tetM* was done by searching against nr nucleotide or protein databases (NCBI, National Center for Biotechnology Information) using megaBLAST^32^ and blastx algorithms with the default parameters^32^,^38^. Chimeras were removed by comparing the consensus sequences to each other (ie. non-nested PCR amplicons to nested IPCR amplicons and pUC19 clones to PacBio libraries) and against the public databases. No PCR chimeras were detected, but chimeras containing two full length amplicons and primer sequences within the read, which can occur in SMRTbell adaptor ligation step of PacBio library preparation^39^ were identified and removed.

Open reading frames were predicted using Prodigal v2.6.136^40^ and annotated using Pannzer^41^ with the default settings. Final annotations were done by combining the nr database hits of the megaBLAST and blastx searches and the automatic annotations of the open reading frames done with Pannzer. The mobile genetic element types were identified based on the annotated genes and sequence identities to mobile genetic elements.

## Additional Information

**How to cite this article**: Pärnänen, K. *et al.* Evaluating the mobility potential of antibiotic resistance genes in environmental resistomes without metagenomics. *Sci. Rep.* **6**, 35790; doi: 10.1038/srep35790 (2016).

## Figures and Tables

**Figure 2:**
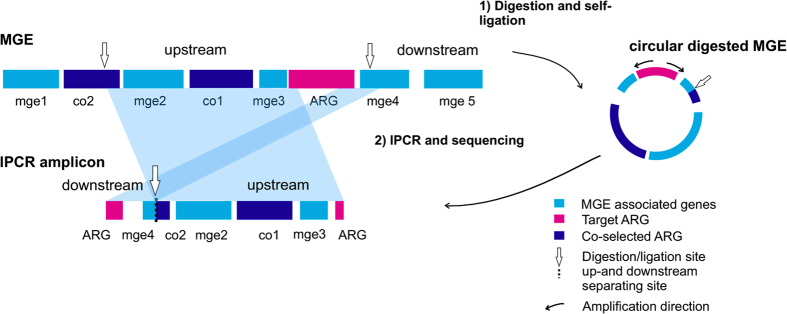
Schematic representation of an IPCR amplicon resulting from digestion, self-circularization and amplification of DNA flanking a resistance gene. Mobile genetic element is digested with restriction enzymes and self-ligated into a circular molecule (1) and amplified (2). The IPCR amplicon contains the up- and downstream regions surrounding the target ARG, which have MGE associated genes (mge1-5) and co-selected ARGs (co1 and co2). The digestion and ligation site separates the up-and downstream regions of the ARG in the IPCR amplicon.

**Table 1:** Illumina Miseq metagenome library statistics.

Year	Replicates	Size (Gbps)	QF reads (Million)	16S rRNA reads	16S rRNA reads/QF reads	*sul1* reads	*tetM* reads	*sul1*/16S rRNA	*tetM*/16S rRNA
2013	3	9.1	17.17	15839	9.2 × 10^−4^	7	35	4.4 × 10^−4^	2.2 × 10^−3^
2009	1	2.2	4.25	1243	2.9 × 10^−4^	1	4	8.0 × 10^−4^	3.2 × 10^−3^
2006	1	2.1	4.17	1099	2.6 × 10^−4^	0	5	—	4.5 × 10^−3^

**Table 2:** Metagenome assembly statistics.

Assembler	Contigs	Max contig length (bp)	Contigs longer than 1500 bp	N50 (min 1500 bp)	Reads mapping to contigs longer than 1500 bp (%)	Year
metaSPADES	2.9 × 10^6^	241 837	6.1 × 10^4^	2636	19.9	2013
metaSPADES	5.6 × 10^5^	13 970	3.7 × 10^3^	1997	3.6	2009
metaSPADES	5.1 × 10^5^	11 779	1.6 × 10^3^	2061	1.7	2006
MEGAHIT	3.1 × 10^6^	38 991	6.3 × 10^4^	2297	20.4	2013
MEGAHIT	5.8 × 10^5^	13 953	1.0 × 10^4^	2015	5.8	2009
MEGAHIT	5.3 × 10^5^	12 057	6.4 × 10^3^	1908	2.6	2006
IDBA-UD	1.3 × 10^6^	79 435	1.0 × 10^5^	2622	22.7	2013
IDBA-UD	1.8 × 10^5^	13 045	6.8 × 10^3^	2188	5.4	2009
IDBA-UD	1.3 × 10^5^	10 326	3.3 × 10^3^	2165	2.9	2006

**Table 3:** Sequences of primers used in IPCR.

Primer	Sequence	Reference
*sul1* inv FW^a^	AAGAACCGCACAATCTCGTC	42
*sul1* inv RV^a^	GGCTTCCGCTATTGGTCTC	42
*sul1* inv nest 1FW^b^	CGAAGAAGGAGTCCTCGGTG	This study
*sul1* inv nest 2FW^b^	GACTCGCAGCATTTCGATCG	This study
*sul1* inv nest 12RV^b^,^c^	TCGGAAACCCTCGCGAAATT	This study
*sul1* inv nest 3FW^b^	CGACATCCACGACGTCTGAT	This study
*sul1* inv nest 3RV^b^	GCCAGAGACCGAGGGTTAGA	This study
*tetM* inv FW^a^	CAGAATTGTTAGAGCCATATC	23
*tetM* inv RV^a^	GCAGAAATCAGTAGAATTGC	23

^a^*sul1* inv FW and RV, and *tetM* inv FW and RV primers have been translated in to reverse complement from previously published primers

^b^Primers used in nested PCR have the word nest attached to the primer name

^c^*sul1* inv nest 12RV was used with both *sul1* inv nest 1FW and 2FW primers as the reverse primer.
